# Exome sequencing identifies variants in two genes encoding the LIM-proteins NRAP and FHL1 in an Italian patient with BAG3 myofibrillar myopathy

**DOI:** 10.1007/s10974-016-9451-7

**Published:** 2016-07-21

**Authors:** Francesca D’Avila, Mirella Meregalli, Sara Lupoli, Matteo Barcella, Alessandro Orro, Francesca De Santis, Clementina Sitzia, Andrea Farini, Pasqualina D’Ursi, Silvia Erratico, Riccardo Cristofani, Luciano Milanesi, Daniele Braga, Daniele Cusi, Angelo Poletti, Cristina Barlassina, Yvan Torrente

**Affiliations:** 1 Department of Health Sciences, Università degli Studi di Milano, via A. Rudinì 8, 20100 Milan, Italy; 2 Department of Pathophysiology and Transplantation, Università degli Studi di Milano, Fondazione IRCCS Ca’ Granda Ospedale Maggiore Policlinico, Centro Dino Ferrari, via F. Sforza, 35, 20122 Milan, Italy; 3 Institute for Biomedical Technologies, CNR, Via Fratelli Cervi N. 93, Segrate, 20090 Milan, Italy; 4 YStem s.r.l., viale Piave 21, 20129 Milan, Italy; 5 Filarete Foundation, Viale Ortles, 22, 20100 Milan, Italy; 6 Dipartimento di Scienze Farmacologiche e Biomolecolari, Centro di Eccellenza per lo studio delle malattie neurodegenerative (CEND), Università degli studi di Milano, via Balzaretti, 9, 20100 Milan, Italy; 7 Department of Health Sciences, Università degli Studi di Milano, c/o Filarete Foundation, Viale Ortles 22/4, 20139 Milan, Italy

**Keywords:** Myofibrillar myopathies, Exome sequencing, LIM proteins, BAG3

## Abstract

Myofibrillar myopathies (MFMs) are genetically heterogeneous dystrophies characterized by the disintegration of Z-disks and myofibrils and are associated with mutations in genes encoding Z-disk or Z-disk-related proteins. The c.626 C > T (p.P209L) mutation in the BAG3 gene has been described as causative of a subtype of MFM. We report a sporadic case of a 26-year-old Italian woman, affected by MFM with axonal neuropathy, cardiomyopathy, rigid spine, who carries the c.626 C > T mutation in the BAG3 gene. The patient and her non-consanguineous healthy parents and brother were studied with whole exome sequencing (WES) to further investigate the genetic basis of this complex phenotype. In the patient, we found that the BAG3 mutation is associated with variants in the NRAP and FHL1 genes that encode muscle-specific, LIM domain containing proteins. Quantitative real time PCR, immunohistochemistry and Western blot analysis of the patient’s muscular biopsy showed the absence of NRAP expression and FHL1 accumulation in aggregates in the affected skeletal muscle tissue. Molecular dynamic analysis of the mutated FHL1 domain showed a modification in its surface charge, which could affect its capability to bind its target proteins. To our knowledge this is the first study reporting, in a BAG3 MFM, the simultaneous presence of genetic variants in the BAG3 and FHL1 genes (previously described as independently associated with MFMs) and linking the NRAP gene to MFM for the first time.

## Introduction

Myofibrillar myopathies (MFMs) are a heterogeneous group of inherited or sporadic neuromuscular disorders with clinical and genetic heterogeneity, characterized by the disintegration of Z disks and myofibrils, followed by the accumulation of myofibrillar degradation products and the ectopic accumulation of multiple proteins in the abnormal fiber regions.

The clinical phenotypes include limb-girdle muscular dystrophy, distal myopathy, scapuloperoneal syndrome or rigid spine syndrome. The diagnosis of MFM is based on clinical findings, electromyography (EMG), nerve conduction studies and, most importantly, muscle histology. Most patients with MFM present progressive muscle weakness but in some patients cardiomyopathy may precede muscle weakness. MFMs follow an autosomal dominant inheritance pattern although X-linked or autosomal recessive inheritance patterns can be occasionally observed.

These disorders have been associated with mutations in genes encoding sarcomeric Z-disk or Z-disk-related proteins, such as desmin (Clemen et al. 2013), alpha crystallin B chain (Vicart et al. 1998), myotilin (Selcen and Engel 2004), Z-band alternatively spliced PDZ motif-containing protein (Selcen and Engel 2005), filamin C (Vorgerd et al. 2005), four and a half LIM domain 1 (Schessl et al. 2008), titin (Pfeffer et al. 2014), Bcl-2-associated athanogene-3 (BAG3) (Selcen et al. 2009). The involvement of BAG3 in MFM has been described in a very limited number of MFM patients who carry the heterozygous mutation c.626 C > T (p.P209L) (Selcen et al. 2009; Odgerel et al. 2010; Jaffer et al. 2012; Lee et al. 2012; Kostera-Pruszczyk et al. 2015). In most of the cases, this mutation has occurred *de novo*. The clinical phenotype includes early age of onset, rapid evolution of the disease, respiratory insufficiency, neuropathy, limb and axial muscle weakness, cardiomyopathy. Rigid spine is present only in a few BAG3 MFM patients. The BAG3 protein belongs to the BAG co-chaperon family and it is involved in major biological processes such as apoptosis, protein quality control, autophagy and cytoskeleton organization (Rosati et al. 2011). Moreover, the BAG3 protein appears to be important for the maintenance of mature skeletal muscle (Homma et al. 2006), although a direct role for BAG3 in muscle function has not yet been fully elucidated (Hishiya et al. 2010).

In this paper, we used whole exome sequencing (WES) to study an Italian female MFM patient who had been previously specifically investigated for mutations in the BAG3 gene and characterized as a carrier of the c.626C > T mutation (Selcen et al. 2009), in order to identify possible other genes involved in the patient’s severe phenotype.

The WES analysis has been extended to her unaffected, non-consanguineous parents and brother. In the patient we identified variants in the NRAP and FHL1 genes that encode muscle-specific, LIM domain containing proteins and investigated their effect at mRNA and protein level in her skeletal muscle. NRAP is a 197 kDa multi-domain scaffolding protein with a N-terminal LIM domain, a C-terminal domain composed of five nebulin-related super-repeats (SR) and a linker region with nebulin-related single repeats (IB) (Mohiddin et al. 2003). We identified three non-synonymous variants in the NRAP gene. Two of them lead to amino acid substitutions in the protein’s SR region, involved in the binding with actin, vinculin (Luo et al. 1999) and filamin C (Lu et al. 2003). The third causes an amino acid change in the IB region, which binds a-actinin (Lu et al. 2003) and MLP (Ehler et al. 2001). FHL1 is a 32 kDa protein characterized by a N-terminal half LIM domain followed by four complete LIM domains. In the FHL1 gene we identified a non-synonymous variant that causes the substitution of an aspartic acid with an asparagine in the fourth LIM domain.

To our knowledge, this is the first study linking variants in the NRAP gene to a MFM phenotype. We also describe the simultaneous occurrence in the same patient of BAG3 and FHL1 gene variants already independently associated to MFMs, and suggest the involvement of BAG3 and FHL1 in the same signaling pathway.

## Materials and methods

### Ethical issue

This study was performed according to the guidelines of the Committee on the Use of Human Subjects in Research of the Policlinico Hospital of Milan (Milan, Italy). Informed consent was obtained from all family members.

### Immunohistochemistry analysis on muscle biopsy

*Triceps surae* muscle biopsies were obtained from healthy subjects and from the patient. Normal subjects were age and sex matched: females aged between 18 and 30 years old. Biopsies were frozen in liquid nitrogen-cooled isopentane and sectioned on a cryostat. Serial sections of 10 µm thickness were stained with Hematoxylin & Eosin, Gomori’s Trichrome, Oil Red O, Acid phosphatase, reduced nicotinamide adenine dinucleotide (NADH), Succinic dehydrogenase (SDH), Cytochrome oxidase, Myofibrillar ATPase and GPD staining. Images were captured using a Leica DM6000B microscope at ×20 and ×40 magnification (Leica, Germany). For immunohistochemical staining, sections were incubated at room temperature for 30 min with a solution of methanol containing 0.03 % H_2_O_2_, for 30 min with 2 % horse serum and then for 1 h with anti-NRAP (1:50, Santa Cruz Biotechnology Inc., Dallas, Texas, USA), anti-FHL1 (1:50, Millipore, Darmstadt, Germany) and anti-BAG3 (1:50, Abcam, Cambridge, UK) antibodies. After rinses with PBS 1X, sections were incubated with biotinylated secondary antibodies (1:100; Vector Laboratories, Burlingame, CA, USA), washed and incubated with the avidin–biotinylated peroxidase complex (avidin-biotin complex method kit, Vector Laboratories, Burlingame, CA, USA). Sections were counterstained with hematoxylin. For all immunostaining, negative controls did not contain the primary antibody, which was replaced with non-immune serum. Images were captured using a Leica DM6000B microscope at 10x and 40x magnification (Leica, Germany).

### Whole exome sequencing (WES)

Genomic DNA was extracted from peripheral venous blood from all family members following standard procedures. For DNA library construction and exome capture, the Agilent SureSelectXT HumanAll Exon 50 Mb kit (Agilent Technologies, Santa Clara, CA, USA) was used starting from 3 µg of genomic DNA. Sequencing was performed as 72 bp paired-end reads on Illumina Genome Analyzer IIx.

The quality check of raw reads was performed using FASTQC (http://www.bioinformatics.babraham.ac.uk/projects/fastqc) and Prinseq (Schmieder and Edwards 011). Reads were aligned using Burrows-Wheeler Aligner BWA (Li and Durbin 2009) with default parameters and using Hg19 as reference genome. We performed duplicate marking, local-realignment around INDELs and base quality score recalibration using Picard Tools (http://picard.sourceforge.net) and the GATK suite (DePristo et al. 2011). We used the GATK Haplotype caller to call single nucleotide variants (SNVs). Annotations were performed using Annovar (Wang et al. 2010) with complete database signatures updated to March 2014. We filtered out variants with low genotype quality (GQ < 50) and coverage lower than 7x. High quality variants underwent a custom prioritization procedure aimed at identifying rare variants at population level in the patient compared to family members: we discarded variants in which the genotype of the patient was homozygous for the reference allele or was identical to the brother or to both parents. We focused on rare non-synonymous and stop-codon variants that followed classical patterns of inheritance (recessive, dominant, compound heterozygous and X-linked). Rarity was set as minor allele frequency (MAF) lower than 1 % in the 1000 Genomes Project. With regard to variants that support a heterozygous compound model of inheritance, rarity was applied when at least one of them followed such rule. Finally, we selected variants mapping in genes mainly expressed in skeletal and cardiac muscle. SIFT (Kumar et al. 2009), CONDEL (González-Pérez and López-Bigas 2011) and PROVEAN (Choi et al. 2012) software were used for the prediction of the pathogenicity of top variants. The genotypes for the variants in the NRAP gene were confirmed in all family members by pyrosequencing using Pyromark Q24 (Qiagen, Valencia, CA, USA). The variant in the FHL1 gene was validated by Sanger sequencing in all family members. All PCR and sequencing primers are listed in Table 1. The genotypes for all the four variants were confirmed in the patient and in her relatives.

**Table 1:** Primer sequences (5′–3′) used for variant validation

Gene	Variation	Validation technique	Forward PCR primer sequence	Reverse PCR primer sequence	Sequencing primer sequence
NRAP	rs200747403	Pyrosequencing	TCCCCACTCATTCAAGTACACA	GCACTTGGAAAGCAAGACTACA	TTATCTGGTTGCTGAATT
NRAP	rs2270182	Pyrosequencing	AAGTACAGGCTGCCTTGTAAAATG	TCCCCACATTGCTCTCTTACCT	CCGTGCTGACTATGAGAA
NRAP	rs2275799	Pyirosequencing	CTGTGCATGGGAGTCAAATTCA	TATTGGTCTGCACATTCCCTTGTC	TCAAGATGCCCTCAG
FHL1	rs151315725	Sanger Sequencing	GTTTCCTCACCTGTATTCATTCAGC	AAATGGGAGAAAAGACGGAAGGAGAAC	GAGAAAAGACGGAAGGAGAAC

### Quantitative PCR analysis (qPCR)

Total RNA was extracted from the biopsy of the patient and of three age and sex matched controls using Trizol Reagent according to the manufacturer’s instructions (Invitrogen Life Technologies, Grand Island, New York, USA). The samples were treated with RNase-Free DNase (Promega, Madison, WI, USA). First strand cDNA was prepared using SuperScript First-Strand III Synthesis System for RT-PCR (Invitrogen Life Technologies), starting from 2 µg total RNA with oligo(dT)_12–18_ primer. Absolute Real-Time PCR (qPCR) was used to have an absolute quantification of human NRAP, FHL1 and BAG3 expression. We also determined the expression of the human-specific GAPDH housekeeping gene for each sample. Primer sequences are shown in Table 2. Target gene levels were measured in real-time with the SYBR GREEN technique using GoTaq MasterMix SyberGreen (Promega, Madison, WI, USA). Each sample was evaluated in triplicate and three independent experiments were performed. Statistical analysis was conducted comparing the average of all experiments by unpaired *T* test (p < 0.05).

**Table 2:** Primer sequences (5′–3′) used in qPCR analysis

Gene	Forward primer sequence	Reverse primer sequence
GAPDH	GTGGCAAAGTGGAGATTGTTGCC	GTAGATGACCCGTTTGGCTCC
BAG3	GCTCCGACCAGGCTACATT	GATAGACATGGAAAGGGTGC
NRAP	GCTGCAGAGTGATGTCAAGTAT	CCGAGCCATTTCCACTTTGTA
FHL1	AAAGGACTGTGTCAAGAGTGAG	AAACAGGGTGAGAGGCAAG

### Western blot analysis

Human muscle biopsies isolated from *Triceps Surae* of three healthy subjects and from the patient were homogenized in a lysis buffer containing 20 mM Tris–HCl (pH 7.8), 140 mM NaCl, 1 mM EDTA, 0.5 % NP40, 1 mM phenylmethylsulfonil fluoride, and complete protease inhibitor mixture (Roche Diagnostics, Rotkreuz, Schweiz), with a POTTER S Homogenizer (B.Braun Biotech International-Sartorius group). Samples were pulsed 5 times for 5 s each at a speed of 1000 rpm. Samples were then passed 5 times through a 30.5-gauge needle to disrupt the nuclei, then incubated at 4 °C for 15 min and finally centrifuged at 13,000 rpm for 15 min at 4 °C. Total protein concentration was determined according to Lowry’s method. Samples were resolved on 8 % polyacrylamide gel for NRAP, 12 % for FHL1 and BAG3 and transferred to nitrocellulose membranes (Bio-Rad Laboratories, Hercules, CA, USA). The following antibodies were used for the assays: anti-NRAP (1:50) (Santa Cruz Biotechnology, Dallas, Texas, USA), anti-FHL1 (1:500) (Millipore, Darmstadt, Germany) and anti-BAG3 (1:1000) (Abcam, Cambridge, UK). The BAG3 antibody recognizes the C terminal part of the protein (196 amino acids). The FHL1 antibody recognizes the amino acid sequence CRDPLQGKKYVQKDGRH (amino acid 10–26).The NRAP antibody recognizes an internal sequence amino 1309–1393 (UniProt ID: Q86VF7). We also determined the expression for each sample of the anti-β-Tubulin III (1:500) (SIGMA, Saint Louis, MO, USA) housekeeping protein. Detection was performed with horseradish peroxidase (HRP)-conjugated secondary antibodies (DakoCytomation, Carpinteria, CA, USA), followed by enhanced chemiluminescence (ECL) development (Amersham Biosciences, Piscataway, NJ, USA). Bands were visualized by autoradiography using Amersham Hyperfilm™ (Amersham Biosciences, Piscataway, NJ, USA). Densitometric analysis was performed using ImageJ software (http://rsbweb.nih.gov/ij/). Each sample was evaluated in three independent experiments.

### Molecular dynamics (MD) simulation and electrostatic potential studies of FHL1 fourth LIM domain

The NMR structure of the fourth LIM domain in FHL1 (PDB ID: 2egq) was obtained from the protein databank (PDB, http://www.rcsb.org/pdb/). Starting from the 3D structure of the fourth LIM domain, the D275N mutant was modeled using SCWRL4 (Krivov et al. 2009). MD simulations of the wild type and D275N mutant were carried out by producing runs of 30 ns using AMBER12. In order to assess the impact of the amino acid substitution on protein folding and on the protein’s ability to bind target proteins, we performed (for the wild type and mutant proteins) a structural statistical analysis of all conformations obtained during molecular dynamics and an electrostatic potential surface analysis using the Adaptive Poisson-Boltzmann Solver program (Baker et al. 2001).

### In vitro wild type and mutated BAG3 transfection experiments

Immortalized human healthy myoblasts (CHQRb) and myoblasts isolated from the patient were expanded and cultured on uncoated standard tissue culture plastic at 37 °C in 5 % CO_2_ 95 % air. Cells were plated onto six-well plastic tissue culture plates in DMEM (Euroclone, Italy), supplemented with 15 % fetal bovine serum (Euroclone, Italy) and 0.1 % penicillin/streptavidin antibiotic. CHQRb cells and the patient’s myoblasts were then seeded at 2.0 × 10^5^ cells per well in a 6-well plate, grown for 24 h and transfected in different experiments with wild type pCIneoHisBag3 or mutated pCIneoHisBag3 c.626C > T. The transfection mixtures for each sample contained 5 µl of Lipofectamine (Invitrogen Life Technologies, Carlsbad, CA, USA) and 3.5 µg plasmid in a 1.4 ml total volume of DMEM (Invitrogen Life Technologies, Carlsbad, CA, USA) without antibiotics and fetal bovine serum. Western blot analysis was performed 48 h after transfection to verify the expression of BAG3, NRAP and FHL1 proteins as described in Materials and Methods. For immunofluorescence analysis, CHQRb transfected cells and the patient’s myoblasts were incubated with primary antibody against FHL1 (1:50) (Millipore, Darmstadt, Germany). Images were captured using the Leica TCS SP2 confocal system (Leica, Germany).

## Results

### Case description

**Fig. 1:**
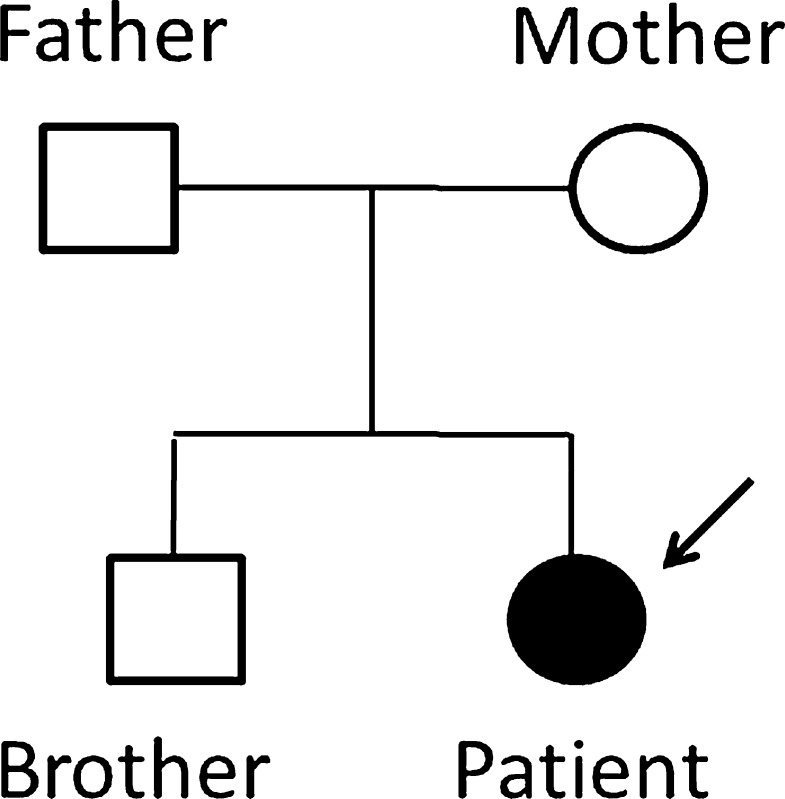
Family pedigree. The patient is marked with an *arrow* and is indicated with a *filled circle*. The unaffected relatives are marked with *open circle*/*square*

We studied an Italian family with a 26-year-old female patient suffering from a BAG3 myopathy, her non-consanguineous asymptomatic healthy parents and her asymptomatic healthy brother (Fig. 1). The age of onset of symptoms in the patient ranged between 11 and 14 years. Creatine kinase (CK) levels ranged from normal to 1.500 U/L. The progression of the disease started with early spinal contractures causing spinal rigidity, scapular winging and later postural muscle atrophy as well as the development of an additional proximal weakness in a limb-girdle distribution pattern with loss of deambulation. Muscle biopsy at age of 13 years showed Z disk aggregates and atrophic type I fibers whereas type II fibers were hypertrophic. Electromyography showed axonal neuropathy. In the last 5 years the patient developed respiratory insufficiency, thus requiring ventilatory support on a continuous basis or overnight. Impaired conduction, arrhythmia and cardiac hypertrophy were reported.

### Characterization of muscle tissue

**Fig. 2:**
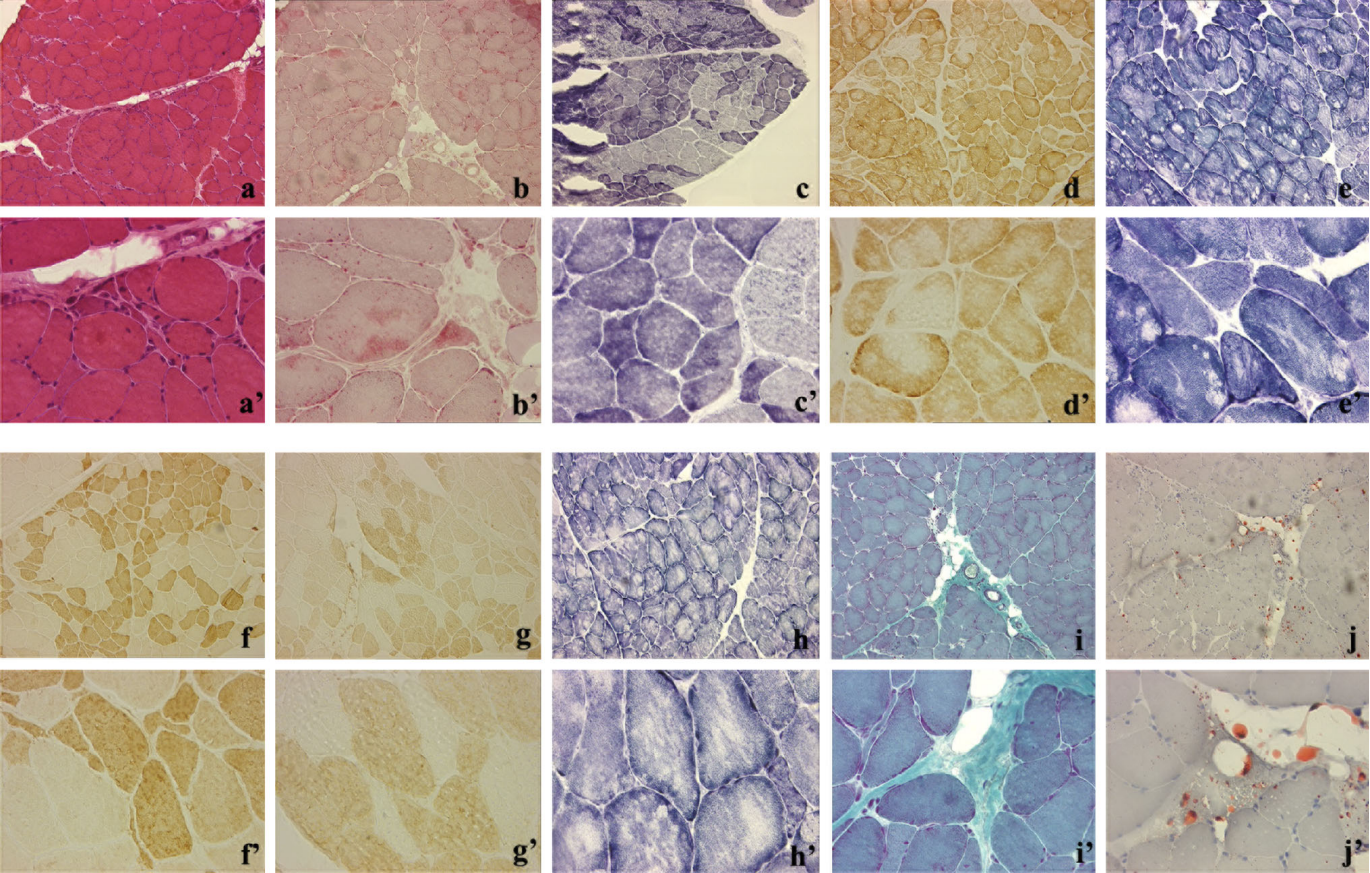
Immunohistochemistry analysis on the patient’s muscle biopsy**. a, a’** Histological characterization of the patient’s muscle tissue by Hematoxylin & Eosin. **b, b’** Acid phosphatase activity showed many cytoplasmic inclusions. **c, c’** Cytoplasmic glycolytic bodies were visualized by αGPD enzyme. Sections were stained for COX (**d, d’**) NADH (**e, e’**), SDH (**h, h’**), showing the presence of amorphous deposits. Fast (**f, f’**) and slow (**g, g’**) myofiber staining was performed. Gomori’s trichrome (**i, i’**) and Oil Red O (**j, j’**) staining. Note the variability in fiber areas, collagen and oil droplets deposition

For this study *Triceps Surae* muscle biopsy was performed on the patient (Fig. 2). All experiments were conducted on the only available dystrophic muscle biopsy. Muscle biopsies from the patient’s parents and brother were not available. The patient’s muscle biopsy showed myofibrillar breakdown and Z-line streaming. Hematoxylin & Eosin staining revealed variability in the diameter of muscle fibers (from 10 to 100 µm), some of them divided by splitting and several muscle fibers in necrosis (Fig. 2a, a’). Some muscle fibers displayed vacuoles. Gomori’s trichrome (Fig. 2i, i’) staining showed increased collagen deposition while Oil Red O staining evidenced the formation of lipid droplets (Fig. 2j, j’). NADH (Fig. 2e, e’), SDH (Fig. 2h, h’) and COX (Fig. 2d, d’) staining showed normal mitochondrial or oxidative metabolism even in the presence of amorphous deposits. Trichrome staining showed small dense granules or amorphous masses in several muscle fibers (data not shown). Conversely, the αGPD enzyme permitted to visualize cytoplasmic glycolytic bodies (Fig. 2c, c’). No difference in the number of fast (Fig. 2f, f’) and slow myofiber staining was observed (Fig. 2g, g’). Increased acid phosphatase activity was evidenced by many cytoplasmic inclusions (Fig. 2b, b’).

### Whole exome sequencing and variant calling

On average 144 million raw 72 bp paired-end reads were generated among the samples, with a mean of 121 million reads after PCR duplicate removal. The mean coverage over the targeted exome across all samples was 46X. After quality filtering and removal of intergenic, intronic and synonymous variants, we identified 3663 SNPs. We confirmed the heterozygous c.626 C > T (p.P209L) mutation in BAG3 in the patient and its absence in her relatives, as previously reported (Selcen et al. 2009). Moreover, both the patient and her relatives were found to be negative for variants in all the already known MFM-related genes such as DES, CRYAB, MYOT, ZASP, FLNC and TTN, except for the FHL1 gene. We identified the non-synonymous variant rs151315725 located in exon 8 (c.823G > A, p.D275N) in the heterozygous FHL1 gene in the patient. The FHL1 gene maps on chromosome X and the patient inherited this variant from her heterozygous mother, in accordance with an X-linked model of inheritance. Neither her father nor her brother carry this variant. Rs151315725 is very rare with a MAF = 0.0048 in the 1000 Genomes Project and is predicted to be deleterious by most prediction tools. After applying all prioritization filters, we also identified three non-synonymous variants in the NRAP gene on chromosome 10: rs200747403 in exon 32 (c.3674G > A, p.A1225 V), rs2270182 in exon 16 (c.1556T > A, p.N519I) and rs2275799 in exon 9 (c.844C > T, p.A282T). Rs200747403 has been annotated in the 1000 Genomes Project with a MAF of the derivative A allele equal to 0.0004. The patient inherited the A allele from her father. At protein level, this variant falls in super domain 4 (SR4) and is predicted to be damaging by most prediction tools. Conversely, the derivative alleles A at rs2270182 and T at rs2275799 are frequent in the 1000 Genomes Project (MAF = 0.25 and 0.27 respectively) and are both inherited from the mother. Rs2270182 falls in SR1 and rs2275799 localizes in the single repeat region (IB). We validated the FHL1 variant in Sanger sequencing and NRAP variants in pyrosequencing. The genotypes for all the four variants were confirmed in the patient and in her relatives.

### BAG3, NRAP and FHL1 expression in muscle biopsies

**Fig. 3:**
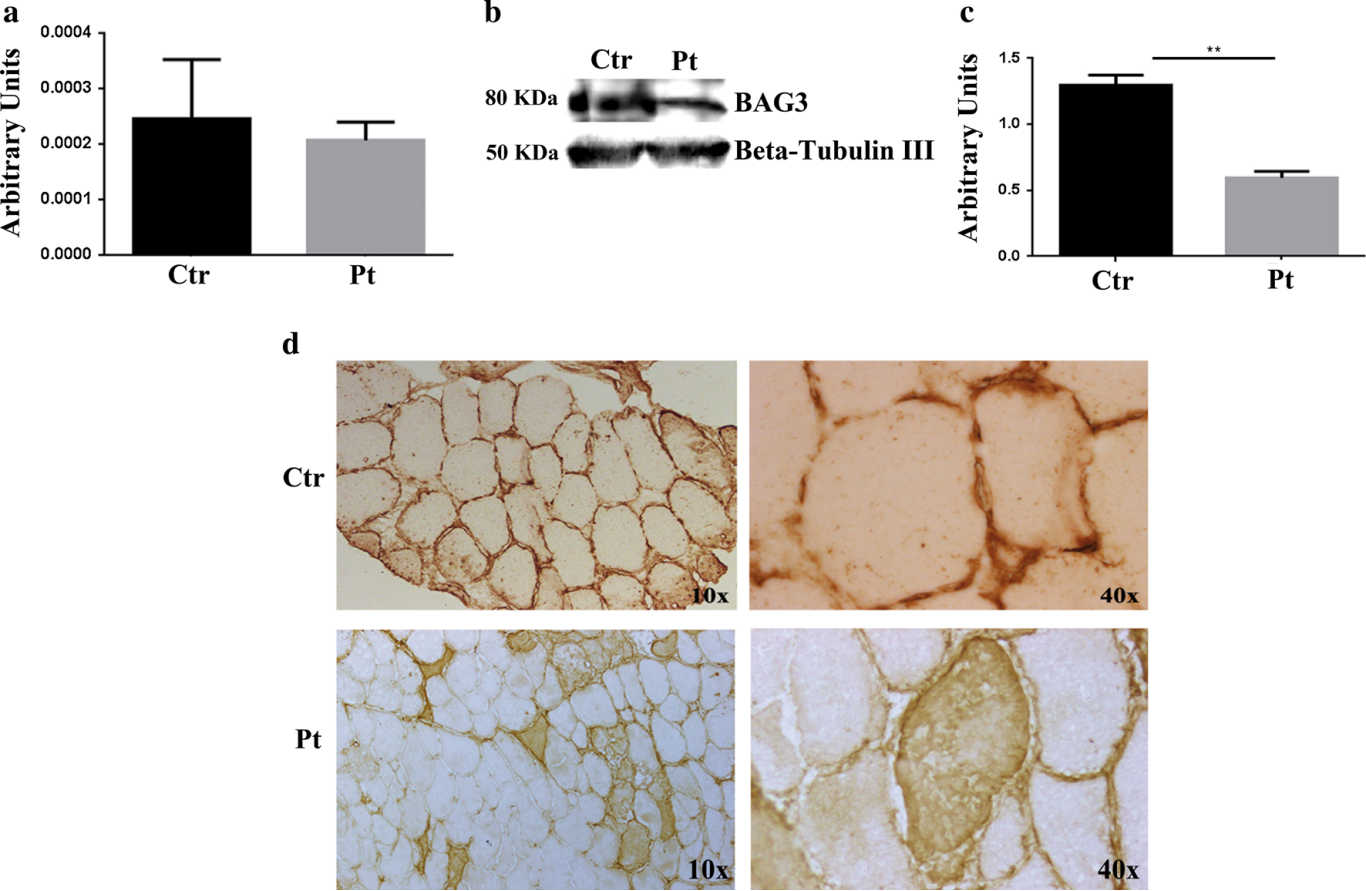
Analysis of BAG3 expression, content and localization in the patient’s muscle biopsy. **a** RT-qPCR analysis of BAG3 mRNA revealed a non-statistically significant downregulation of BAG3 expression in the patient’s muscle compared with healthy controls. **b** WB analysis of BAG3 performed on the patient’s and control muscles showed a downregulation of BAG3 expression in the patient’s muscle. Images of bands were obtained using the CanoScan LiDE60 Scanner (Canon) and the Canon ScanGear Software. **c** Densitometric analysis of the protein levels was performed using ImageJ software (http://rsbweb.nih.gov/ij/). **d** Immunohistochemical analysis of BAG3 in the patient’s and control muscles. In the controls BAG3 localized with sarcolemma, while in the patient it accumulated in the cytoplasm of some muscle fibers

**Fig. 4:**
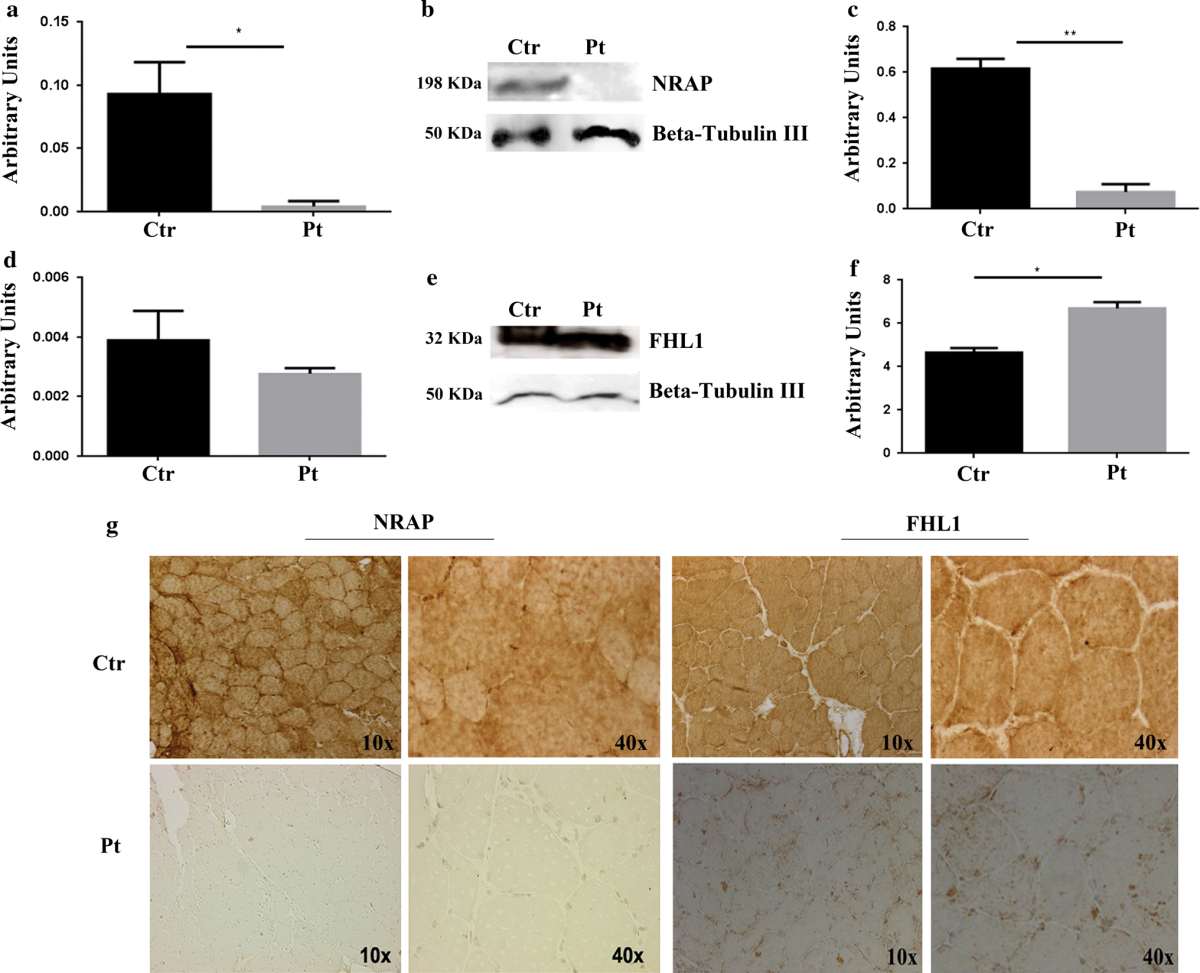
Analysis of NRAP and FHL1 expression, content and localization in the patient’s muscle biopsy. **a** RT-qPCR analysis showed a statistically significant downregulation of NRAP expression in the patient’s muscle compared with healthy controls (unpaired *t* test, p < 0.05). **b** WB analysis of NRAP in the patient’s and control muscle. The analysis showed the absence of NRAP expression in the patient. **c** Densitometric analysis of the protein levels was performed using ImageJ software (http://rsbweb.nih.gov/ij/). **d** RT-qPCR analysis showed a non-statistically significant downregulation of FHL1 in the patient’s muscle compared to healthy controls. **e** WB analysis revealed an overexpression of FHL1 in the patient’s muscle compared to the healthy controls. **f** Densitometric analysis of the protein levels was performed using ImageJ software (http://rsbweb.nih.gov/ij/). **g** Immunohistochemical analysis of NRAP was performed on the patient’s and control muscles. In the control NRAP localized with myofibrils, while in the patient it was not detectable. Immunohistochemical analysis of FHL1 demonstrated an intracytoplasmatic myofiber association of the protein in the controls while in the patient it was detectable in intracytoplasmatic aggregates

All experiments were conducted on the only available dystrophic muscle biopsy from the patient and on three healthy muscle tissues. Muscle biopsies from the healthy patient’s parents and brother were not available. Real time PCR results showed a non-statistically significant reduction of BAG3 (Fig. 3a) and a statistically significant reduction of NRAP mRNA expression in the patient (p < 0.05) (Fig. 4a), while FHL1 mRNA tended to be lower in the patient though the difference was not statistically significant (Fig. 4d). Western Blot analysis confirmed that the levels of BAG3 (Fig. 3b, c) and NRAP (Fig. 4b, c) proteins in the patient’s muscle were respectively reduced and absent compared to the healthy muscle. Moreover, we demonstrated an increased expression of FHL1 protein isoform A (Fig. 4e, f) and the absence of the other isoforms compared to the control muscle specimens (data not shown). Immunohistochemistry (IHC) analysis was performed in the patient’s and control muscle to evaluate BAG3, NRAP and FHL1 localization. In the patient, BAG3 (Fig. 3d) was present in abnormal cytoplasmic accumulations in some myofibers, whereas NRAP (Fig. 4g) was totally absent. On the contrary, subsarcolemmal and intra-cytoplasmic immunoreactivity of FHL1 was observed in several muscle fibers suggesting the presence of FHL1 in aggregates (Fig. 4g).

### Molecular dynamics (MD) simulation and electrostatic potential studies of fourth LIM domain of FHL1

**Fig. 5:**
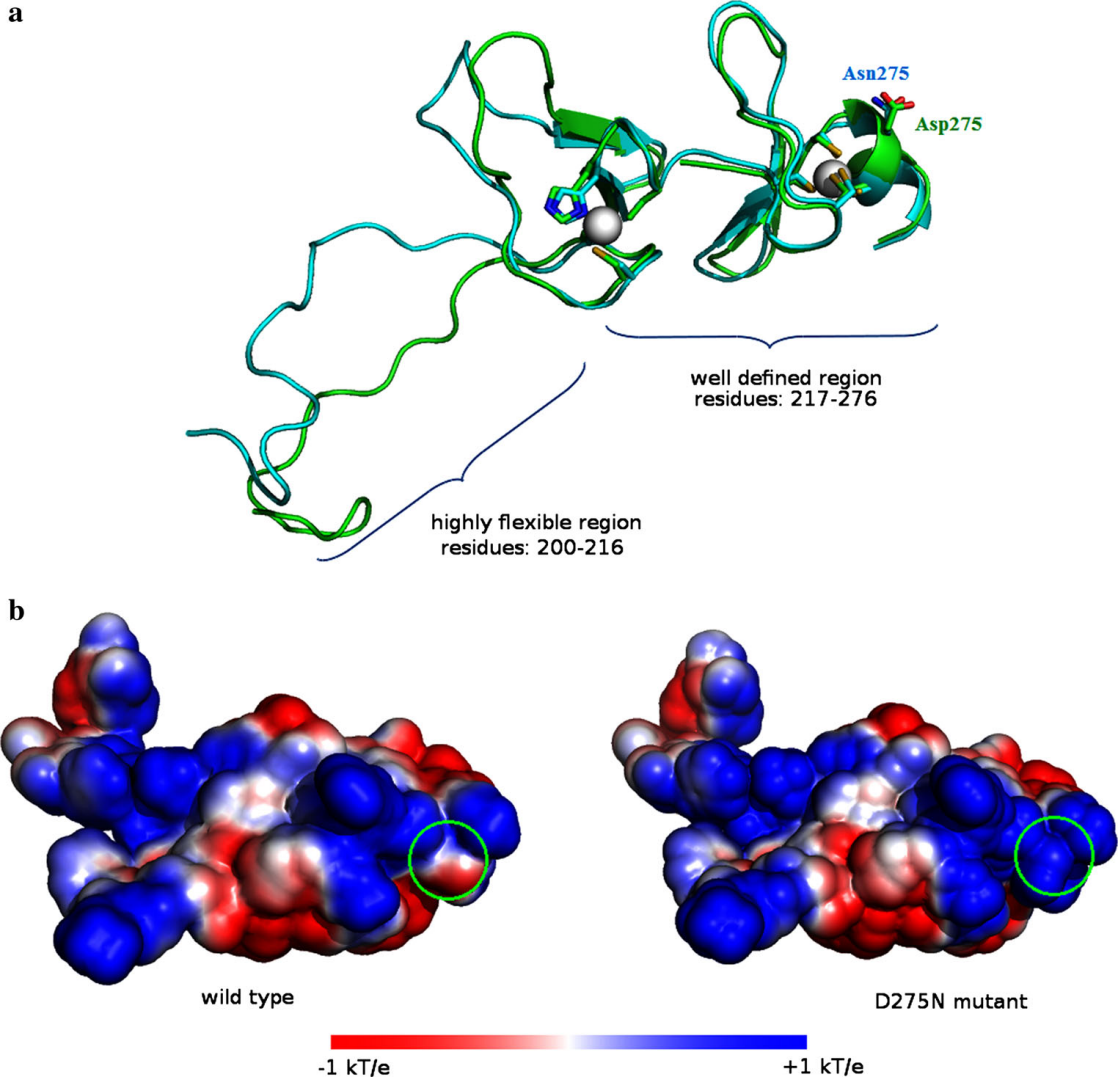
Molecular dynamics simulation and electrostatic potential study of FHL1 fourth LIM domain. **a** Structure of fourth LIM domain from MD simulation of wild-type FHL1 and the D275N FHL1 mutant. Each structure was the most representative frame obtained from the cluster analysis of the simulations. A ribbon representation of the wild-type protein is shown in *green* with the mutant structure superimposed in cyan. Asp275, Asn275 mutant and the zinc ion coordination residues are shown in rod and *colored* by atom type, while the zinc ions are shown in space-filling (van der Waals) representations. The D275N mutant remained folded in native conformation, with zinc sites almost fully intact. **b** Surface electrostatic potential distribution of D275N mutant compared to the wild type LIM domain. Green circles indicate the position of Asp275 and Asn275 in wild type and mutant, respectively. The potential scale ranges from −1 kT/e to 1 kT/e from *red* to *blue*

MD simulations of the fourth LIM domain were carried out to investigate the relationship between structure and function in the D275N mutant. A statistical analysis (clustering) of the structures was performed to obtain the most representative of both wild type and mutant for their comparison. NMR data (pdb ID:2egq) showed that the FHL1 wild type adopted a well-defined structure (residues 217–276) and a highly flexible region (residues 200–216). Wild type and mutant protein showed very similar representative structures in the well-defined region (residues 217–276), suggesting conservation of the overall tertiary structure of the fourth LIM domain in the presence of the amino acid substitution (Fig. 5a). The structural analysis of the second Zinc atom involved in the C-terminal binding site conserved the coordination status during the simulation in spite of the proximity with the mutated residue (275N). On the other hand, the mutated 275N residue located on the surface of the domain modified the local charge of the surface compared to the wild type. To evaluate the effect of charge modification on protein function due to the D275N change, the electrostatic potential on the fourth LIM domain surface was calculated. It was observed that the D275N change was responsible for a modification of the surface charge distribution in the C-terminal Zinc binding site. The replacement of aspartic acid with an asparagine results in a generalized positivization of the surface area (Fig. 5b).

### In vitro evaluation of NRAP and FHL1 expression in human normal myoblasts and in the patient’s myoblasts

**Fig. 6:**
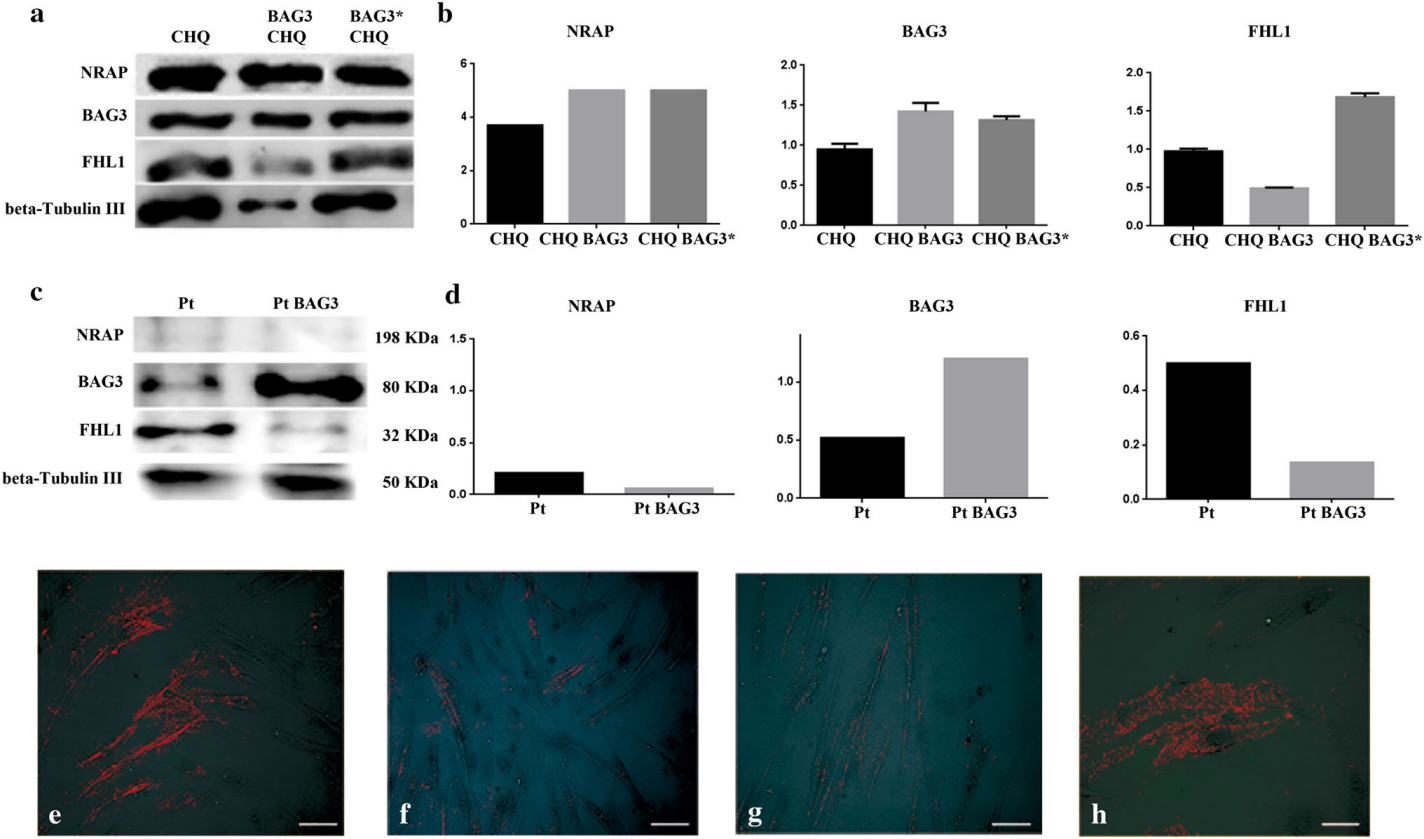
In vitro evaluation of NRAP and FHL1 expression after transfection with wild type and mutated BAG3. **a** Evaluation of the expression of BAG3, NRAP and FHL1 expression in CHQRb cells before and after transfection with wild-type pCIneoHisBag3 (BAG3) and mutated pCIneoHisBag3 c626C > T (BAG3*) by WB analysis. **b** Densitometric analysis of BAG3, NRAP and FHL1 levels in CHQRb cells performed using ImageJ software (http://rsbweb.nih.gov/ij/). **c** WB analysis for BAG3, NRAP and FHL1 expression in patient’s myoblasts before and after transfection with wild-type pCIneoHisBag3 (BAG3). **d** Densitometric analysis of BAG3, NRAP and FHL1 levels in patient’s myoblasts performed using ImageJ software (http://rsbweb.nih.gov/ij/). **e–h** Evaluation of the expression of FHL1 in patient’s myoblasts before (**e**) and after (**f**) transfection with wild type pCIneoHisBag3 (BAG3) and in normal myoblasts treated with wild type (**g**) and mutated (**h**) BAG3 by immunofluorescence analysis. Images were captured using the Leica TCS SP2 confocal system (Leica, Germany), ×20 magnification, scale bar 100 μm

In order to evaluate the influence of the c.626C > T BAG3 mutation on NRAP and FHL1 expression, we performed transfection experiments in immortalized human healthy myoblasts (CHQRb) and in the patient’s myoblasts. CHQRb cells were transfected with wild type BAG3 (pCIneoHisBag3) or the mutated form of BAG3 (pCIneoHisBag3 c.626C > T) (BAG3^P209L^), and NRAP and FHL1 expression was evaluated by Western blot. No differences in NRAP expression were detected in CHQRb cells transfected with wild type BAG3 or BAG3^P209L^ (Fig. 6a, b). These results suggest that the absence of mRNA (Fig. 4a) and protein (Fig. 4b, c) found in the patient’s muscle was not influenced by mutated BAG3. On the contrary, FHL1 expression was reduced in CHQRb overexpressing wild type BAG3, and it was increased in CHQRb overexpressing BAG3^P209L^ (Fig. 6a, b) compared to the control. The patient’s myoblasts were transfected only with wild type BAG3 and no difference in NRAP expression was detected compared to non-transfected patient myoblasts (Fig. 6c, d). On the contrary, FHL1 expression was reduced in the patient’s myoblasts transfected with wild type BAG3 compared to non-transfected patient myoblasts (Fig. 6c, d), similarly to what we observed in CHQRb transfected with wild type BAG3 (Fig. 6a, b).

Since these data suggest that BAG3 influences FHL1 expression both in CHQRb and in the patient’s myoblasts, we investigated whether BAG3^P209L^ affects FHL1 aggregation in these cell types. When wild type BAG3 was expressed in CHQRb cells, FHL1 had a diffuse cytosolic localization (Fig. 6g). In contrast, CHQRb cells expressing BAG3^P209L^ showed a fine granular FHL1 positive pattern with significant perinuclear aggregation (Fig. 6h). Moreover, FHL1 expression was reduced in the patient’s myoblasts transfected with wild type BAG3 (Fig. 6f) compared with the patient’s non-transfected myoblasts (Fig. 6e).

## Discussion

In this work we provide data showing the co-presence of genetic variants in the NRAP and FHL1 genes in a patient with phenotypic features of MFM and who carries the BAG3 c.626C > T mutation. The MFM BAG3 phenotype is very rare and accounts for twelve patients described worldwide. In this paper we focused on the only known Italian case of Bag3Opathy and examined the patient and her unaffected relatives with WES. WES received widespread consideration for the discovery of novel causative variants also in small pedigrees (Glazov et al. 2011). The genetic variants identified in the patient map in the NRAP and FHL1 genes, which are specifically expressed in muscles and both encode LIM domain containing proteins. LIM domain containing proteins have been reported as important for normal skeletal and cardiac structure and function. Mutations in MLP (muscle LIM protein) and in Cipher/ZASP have been found in patients affected by both dilated and hypertrophic cardiomyopathy (Knöll et al. 2002; Geier et al. 2003; Sheikh et al. 2007) and Cypher/ZASP mutations were also identified in zaspopathies, a subtype of MFM (Selcen and Engel 2005; Griggs et al. 2007).

NRAP is an actin-binding LIM protein encoded by a gene mapping on chromosome 10q25 (Luo et al. 1997a, b) and specifically expressed in skeletal and cardiac muscle tissues (Mohiddin et al. 2003; Luo et al. 1997a, b). In the patient, we identified three non-synonymous variants in the NRAP gene: rs200747403 (inherited from her father and predicted as deleterious), rs2270182 and rs2275799, inherited from her mother and predicted as benign. Rs200747403 and rs2270182 localize in the SR region, which contains the binding sites for actin, vinculin (Luo et al. 1999) and filamin C (Lu et al. 2003). Rs2275799 falls in the IB region that binds a-actinin (Lu et al. 2003) and MLP (Ehler et al. 2001). In the patient, both alleles of the NRAP gene are affected by variants that cause the substitution of highly conserved amino acids and NRAP was completely absent at mRNA and protein level in the patient’s muscle. The absence of NRAP mRNA in the patient’s muscle suggests that both the very rare rs200747403 inherited from the father and the variants rs2270182 and rs2275799 inherited from the mother may have a deleterious effect on the messenger’s stability by affecting both NRAP alleles. Though mRNA instability has been linked to the presence of non-sense and frame-shift variants in previous papers (Gong et al. 2007; Zarraga et al. 2011), Vasilopoulos et al. have also reported a decreased mRNA stability caused by a non-synonymous substitution (Vasilopoulos et al. 2007). Our transfection experiments suggest that the absence of NRAP in the patient’s muscle may be due to the three variants and not to the BAG3 mutated allele. NRAP mRNA and protein levels in the patient’s relatives were not evaluated because no biopsies were available. To our knowledge, this is the first study describing variants in the NRAP gene in a MFM phenotype. The absence of NRAP protein in the patient’s muscle could contribute to the disorganized myofibril assembly (Manisastry et al. 2008; Carroll et al. 2004). Dhume et al. were able to demonstrate that NRAP knockdown in cultured embryonic mouse cardiomyocytes resulted in disorganized myofibril assembly and led to a decrease in non-muscle myosin (NMHC) IIB by post-transcriptional mechanisms (Dhume et al. 2006). Moreover, there are reports of a direct interaction of NRAP with NMHC IIB and a decrease of NRAP protein levels in mouse cardiomyocytes by NMHC IIB knockdown (Lu and Horowits 2008). The Authors assigned two separate but tightly linked functional roles in cardiomyocyte biology to NMHC IIB and NRAP, with NMHC IIB involved in cardiomyocyte spreading and NRAP in myofibril assembly. However, the role of NMHC IIB in the assembly of pre-myofibrils is still questioned and to be elucidated (Lu and Horowits 2008; Tullio et al. 1997). No variant in the NMHC IIB (MYH10) gene was found either in the patient or in her relatives.

We also identified the c823G > A transition (rs151315725) in the FHL1 gene, already reported in the 1000 Genomes Project, although extremely rare (MAF = 0.0048), leading to D275N amino acid substitution in one of the three splicing isoforms of the FHL1 protein, i.e. FHL1A, which is the full-length protein (rewieved in Cowling et al. 2011). Rs151315725 has been already reported as a variant in a German family with the characteristic XMPMA phenotype and the Authors hypothesized a negative effect of the D275N amino acid change on FHL1 function when inherited together with the V280 M amino acid substitution caused by the c.838G > A missense mutation (Schoser et al. 2009). Moreover, rs151315725 was also identified in three unrelated European families with hypertrophic cardiomyopathy (HCM), and FHL1^D275N^ was found to activate a fetal hypertrophic gene program in rat-engineered heart tissue, suggesting that it could cause hypertrophic cardiomyopathy (HCM) in these patients (Friedrich et al. 2012). Mutations in the FHL1 gene have been also associated with arrhythmias, HCM and dilated cardiomyopathy in several patients affected by skeletal muscle disorders as well (reviewed in Cowling et al. 2011). Rs151315725 could play a role in the patient’s compromised cardiac phenotype, which includes arrhythmias and cardiac hypertrophy. Additionally, rs151315725 has been reported as deleterious in the NHLBI Exome Sequencing Project (ESP) (https://esp.gs.washington.edu/drupal/) (MAF = 0.011) and in the Exome Aggregation Consortium (ExAC) (http://exac.broadinstitute.org/) (MAF = 0.013) databases, both including individuals affected by heart diseases. This could explain the difference in MAF reported in these databases compared to the 1000 Genomes Project. In addition to XMPMA (Windpassinger et al. 2008), mutations in the FHL1 gene have been associated with other four clinically distinct human myopathies, including reducing body myopathy (RBM) (Schessl et al. 2008, 2009), X-linked dominant scapuloperoneal myopathy (SPM) (Quinzii et al. 2008; Chen et al. 2010), Emery-Dreifuss muscular dystrophy (EDMD) (Gueneau et al. 2009), and rigid spine syndrome (RSS) (Shalaby et al. 2008). Spinal rigidity is the most common clinical feature associated with FHL1 mutations and has been reported in patients with RBM (Schessl et al. 2010), XMPMA (Schoser et al. 2009) and EDMD (Gueneau et al. 2009). In our patient rs151315725 in the FHL1 gene is associated with a BAG3 MFM phenotype that also presents the rigid spine feature.

Rs151315725 in FHL1 is present in the patient’s asymptomatic mother and this could be explained by X chromosome inactivation and by the mosaic for X-linked gene expression in heterozygous women. As previously reported for members of a German family affected by a familiar reducing body myopathy, females carrying the C150R amino acid change in the FHL1 LIM2 domain showed various clinical manifestations and may be asymptomatic, reflecting different degrees of X-inactivation (Schessl et al. 2010). We did not perform any X-inactivation study in this family. We further investigated mRNA and protein expression in the patient’s muscle biopsy and found a slight (though not statistically significant) reduction in mRNA and a weak increase in FHL1 isoform expression, which could be linked to FHL1 accumulation in the deposits observed in the patient’s muscle fibers. Additionally, in the patient’s myoblasts we observed a threefold increase in FHL1 protein expression compared to normal myoblasts (data not shown).

In order to assess the effect of the D275N change on FHL1 function, MD simulation and electrostatic potential analysis were performed on the fourth LIM domain of the protein. MD simulations showed that the wild type and the mutant had the same structural regions and that the second zinc binding site remained largely intact in the mutant. These findings suggest that protein function is not impaired by structural distortions that could generate unfolded protein. In line with these data, gene transfer experiments showed that the FHL1^D275N^ protein was stable in cardiac myocytes and the protein levels were not affected by proteasome inhibition (Friedrich et al. 2012). Electrostatic potential studies showed the loss of negative potential on the fourth LIM domain surface that might create an unfavorable environment for the recognition of FHL1 target proteins. These data are supported by the observation that the FHL1^D275N^ protein had a lower binding specificity for proteins localized at the I-band (Friedrich et al. 2012; Sheikh et al. 2008). Indeed, FHL1A localizes to the sarcomeric I-band and to focal adhesions (Brown et al. 1999; Ng et al. 2001). Little is known about FHL1 function, but the available data suggest that it participates in muscle growth and differentiation, as well as in the assembly of the sarcomere, and that it is a regulator of skeletal muscle mass through the interaction with transcription factors and myosin-binding protein C (Cowling et al. 2008; McGrath et al. 2006). Domenighetti et al. demonstrated that FHL1-null mice develop an age-dependent myopathy associated with myofibrillar and intermyofibrillar disorganization (Domenighetti et al. 2014). FHL1 was recently found as part of a complex which binds gamma-actin and NMHC IIB in vivo and in vitro (Wang et al. 2013). Both NRAP and FHL1 are partners of NMHC IIB (Lu and Horowits 2008; Wang et al. 2013) and the patient’s variants in these genes could affect their interactions with NMHC IIB, with possible implications in myofibrillar assembly.

Our study started from the identification in the patient of the P209L mutation in the BAG3 gene (Selcen et al. 2009). In the original paper by Selcen, the authors described a subtype of MFM sharing common clinical features such as progressive limb and axial muscle weakness, contractures, respiratory insufficiency and hypertrophic cardiomyopathy (Selcen et al. 2009). A characteristic of the disease is the association with peripheral neuropathy that may in some patients be the initial clinical manifestation (Jaffer et al. 2012). The morphological analysis performed in the patient confirmed the feature of MFM.

BAG3 is co-chaperone for other heat shock proteins and has anti-apoptotic properties. It localizes to and co-chaperones the Z disk in skeletal and cardiac muscles. BAG3 protein deficiency determines fulminant myopathy and early mortality in mice (Homma et al. 2006) and BAG3^P209L^ tends to aggregate into small granules, probably as a result of its altered folding and function (Selcen et al. 2009). The results of our study demonstrated a lower muscular expression of BAG3 compared to the healthy controls, both at mRNA and protein level. In the patient, BAG3 IHC showed cytoplasmic accumulation in some muscle fibers. This data could be explained by a mechanism of wild type BAG3 sequestration into BAG3^P209L^ mediated aggregates (Ruparelia et al. 2014), leading to a different localization or rapid degradation of this protein. In particular, the replacement of the 209 leucine caused by the BAG3 mutation in the patient could affect the binding properties of the wild type BAG3 domains.

Our transfection experiments show that BAG3 influences FHL1 protein content both in CHQRb and in the patient’s myoblasts, suggesting the involvement of these two proteins in the same signaling pathway. To address this, Feldkirchner et al. used a quantitative proteomic approach to explore the plaque content in a MFM patient carrying the C224W amino acid substitution in FHL1 and found the accumulation of a series of 15 proteins including FHL1, BAG3 and NRAP (Feldkirchner et al. 2012).

The effect of wild type BAG3 transfected in the patient’s myoblasts suggests a role for wild type BAG3 in the control of FHL1 protein turnover, while this does not occur with the mutated BAG3 form.

Immunofluorescence analysis shows that wild type BAG3 expression in CHQRb and in the patient’s myoblasts gives rise to a diffuse cytosolic immuno-localization of FHL1, whereas both BAG3^P209L^ expressed in CHQRb and the endogenous BAG3^P209L^ in the patient’s myoblasts caused a prevalently perinuclear immuno-localization of FHL1. These data suggest that the different localization and level of aggregation of FHL1 depend on wild type BAG3 form.

In conclusion, this is the first study linking variants in the NRAP gene to a MFM phenotype and reporting, in the same patient, the simultaneous occurrence of BAG3 and FHL1 gene variants that have already been independently associated with MFMs. Moreover, our data suggest that BAG3 and FHL1 could be involved in the same signaling pathway. These data could also suggest that few genes might modulate this MFM phenotype. Examples are present in the literature revealing that for some diseases the phenotype cannot be completely explained by mutations at a single locus. This also applies to some neuromuscular disorders (Badano and Katsanis 2002; Cady et al. 2015; Van Blitterswijk et al. 2012). The aetiology of these diseases could then require the combined action of mutant alleles at a small set of genes.

## References

[CR1] Badano JL, Katsanis N (2002). Beyond Mendel: an evolving view of human genetic disease transmission. Nat Rev Genet.

[CR2] Baker NA, Sept D, Joseph S, Holst MJ, McCammon JA (2001). Electrostatics of nanosystems: application to microtubules and the ribosome. Proc Natl Acad Sci USA.

[CR3] Brown S, McGrath MJ, Ooms LM, Gurung R, Maimone MM, Mitchell CA (1999). Characterization of two isoforms of the skeletal muscle LIM protein 1, SLIM1. Localization of SLIM1 at focal adhesions and the isoform SLIMMER in the nucleus of myoblasts and cytoplasm of myotubes suggests distinct roles in the cytoskeleton and in nuclear. J Biol Chem.

[CR4] Cady J, Allred P, Bali T, Pestronk A, Goate A, Miller TM, Mitra RD, Ravits J, Harms MB, Baloh RH (2015). Amyotrophic lateral sclerosis onset is influenced by the burden of rare variants in known amyotrophic lateral sclerosis genes. Ann Neurol.

[CR5] Carroll S, Lu S, Herrera AH, Horowits R (2004). N-RAP scaffolds I-Z-I assembly during myofibrillogenesis in cultured chick cardiomyocytes. J Cell Sci.

[CR6] Chen D, Raskind WH, Parson WW, Sonnen JA, Vu T, Zheng Y, Matsushita M, Wolff J, Lipe H, Bird TD (2010). A novel mutation in FHL1 in a family with X-linked scapuloperoneal myopathy: phenotypic spectrum and structural study of FHL1 mutations. J Neurol Sci.

[CR7] Choi Y, Sims GE, Murphy S, Miller JR, Chan AP (2012). Predicting the functional effect of amino acid substitutions and indels. PLoS One.

[CR8] Clemen CS, Herrmann H, Strelkov SV, Schröder R (2013). Desminopathies: pathology and mechanisms. Acta Neuropathol.

[CR9] Cowling BS, McGrath MJ, Nguyen MA, Cottle DL, Kee AJ, Brown S, Schessl J, Zou Y, Joya J, Bönnemann CG, Hardeman EC, Mitchell CA (2008). Identification of FHL1 as a regulator of skeletal muscle mass: implications for human myopathy. J Cell Biol.

[CR10] Cowling BS, Cottle DL, Wilding BR, D’Arcy CE, Mitchell CA, McGrath MJ (2011). Four and a half LIM protein 1 gene mutations cause four distinct human myopathies: a comprehensive review of the clinical, histological and pathological features. Neuromuscul Disord.

[CR11] DePristo MA, Banks E, Poplin R, Garimella KV, Maguire JR, Hartl C, Philippakis AA, del Angel G, Rivas MA, Hanna M, McKenna A, Fennell TJ, Kernytsky AM, Sivachenko AY, Cibulskis K, Gabriel SB, Altshuler D, Daly MJ (2011). A framework for variation discovery and genotyping using next- generation DNA sequencing data. Nat Genet.

[CR12] Dhume A, Lu S, Horowits R (2006). Targeted disruption of N-RAP gene function by RNA interference: a role for N-RAP in myofibril organization. Cell Motil Cytoskelet.

[CR13] Domenighetti AA, Chu PH, Wu T, Sheikh F, Gokhin DS, Guo LT, Cui Z, Peter AK, Christodoulou DC, Parfenov MG, Gorham JM, Li DY, Banerjee I, Lai X, Witzmann FA, Seidman CE, Seidman JG, Gomes AV, Shelton GD, Lieber RL, Chen J (2014). Loss of FHL1 induces an age-dependent skeletal muscle myopathy associated with myofibrillar and intermyofibrillar disorganization in mice. Hum Mol Genet.

[CR14] Ehler E, Horowits R, Zuppinger C, Price RL, Perriard E, Leu M, Caroni P, Sussman M, Eppenberger HM, Perriard JC (2001). Alterations at the Intercalated disk associated with the absence of muscle LIM protein. J Cell Biol.

[CR15] Feldkirchner S, Schessl J, Müller S, Schoser B, Hanisch FG (2012). Patient-specific protein aggregates in myofibrillar myopathies: laser microdissection and differential proteomics for identification of plaque components. Proteomics.

[CR16] Friedrich FW, Wilding BR, Reischmann S, Crocini C, Lang P, Charron P, Müller OJ, McGrath MJ, Vollert I, Hansen A, Linke WA, Hengstenberg C, Bonne G, Morner S, Wichter T, Madeira H, Arbustini E, Eschenhagen T, Mitchell CA, Isnard R, Carrier L (2012). Evidence for FHL1 as a novel disease gene for isolated hypertrophic cardiomyopathy. Hum Mol Genet.

[CR17] Geier C, Perrot A, Ozcelik C, Binner P, Counsell D, Hoffmann K, Pilz B, Martiniak Y, Gehmlich K, van der Ven PF, Fürst DO, Vornwald A, von Hodenberg E, Nürnberg P, Scheffold T, Dietz R, Osterziel KJ (2003). Mutations in the human muscle LIM protein gene in families with hypertrophic cardiomyopathy. Circulation.

[CR18] Glazov E, Zankl A, Donskoi M, Kenna TJ, Thomas GP, Clark GR, Duncan EL, Brown MA (2011). Whole-exome re-sequencing in a family quartet identifies POP1 mutations as the cause of a novel skeletal dysplasia. PLoS Genet.

[CR19] Gong Q, Zhang L, Vincent GM, Horne BD, Zhou Z (2007). Nonsense mutations in hERG cause a decrease in mutant mRNA transcripts by nonsense-mediated mRNA decay in human long-QT syndrome. Circulation.

[CR20] González-Pérez A, López-Bigas N (2011). Improving the assessment of the outcome of nonsynonymous SNVs with a consensus deleteriousness score, Condel. Am J Hum Genet.

[CR21] Griggs R, Vihola A, Hackman P, Talvinen K, Haravuori H, Faulkner G, Eymard B, Richard I, Selcen D, Engel A, Carpen O, Udd B (2007). Zaspopathy in a large classic late-onset distal myopathy family. Brain.

[CR22] Gueneau L, Bertrand AT, Jais JP, Salih MA, Stojkovic T, Wehnert M, Hoeltzenbein M, Spuler S, Saitoh S, Verschueren A, Tranchant C, Beuvin M, Lacene E, Romero NB, Heath S, Zelenika D, Voit T, Eymard B, Ben Yaou R, Bonne G (2009). Mutations of the FHL1 gene cause emery-dreifuss muscular dystrophy. Am J Hum Genet.

[CR23] Hishiya A, Kitazawa T, Takayama S (2010). BAG3 and Hsc70 interact with actin capping protein CapZ to maintain myofibrillar integrity under mechanical stress. Cir Res.

[CR24] Homma S, Iwasaki M, Shelton GD, Engvall E, Reed JC, Takayama S (2006). BAG3 deficiency results in fulminant myopathy and early lethality. Am J Pathol.

[CR25] Jaffer F, Murphy SM, Scoto M, Healy E, Rossor AM, Brandner S, Phadke R, Selcen D, Jungbluth H, Muntoni F, Reilly MM (2012). BAG3 mutations: another cause of giant axonal neuropathy. J Peripher Ner Syst.

[CR26] Knöll R, Hoshijima M, Hoffman HM, Person V, Lorenzen-Schmidt I, Bang ML, Hayashi T, Shiga N, Yasukawa H, Schaper W, McKenna W, Yokoyama M, Schork NJ, Omens JH, McCulloch AD, Kimura A, Gregorio CC, Poller W, Schaper J, Schultheiss HP, Chien KR (2002). The cardiac mechanical stretch sensor machinery involves a Z disc complex that is defective in a subset of human dilated cardiomyopathy. Cell.

[CR27] Kostera-Pruszczyk A, Suszek M, Płoski R, Franaszczyk M, Potulska-Chromik A, Pruszczyk P, Sadurska E, Karolczak J, Kamińska AM, Rędowicz MJ (2015). BAG3-related myopathy, polyneuropathy and cardiomyopathy with long QT syndrome. J Muscle Res Cell Motil.

[CR28] Krivov GG, Shapovalov MV, Dunbrack RL (2009). Improved prediction of protein side-chain conformations with SCWRL4. Proteins.

[CR29] Kumar P, Henikoff S, Ng PC (2009). Predicting the effects of coding non-synonymous variants on protein function using the SIFT algorithm. Nat Protoc.

[CR30] Lee HC, Cherk SW, Chan SK, Wong S, Tong TW, Ho WS, Chan AY, Lee KC, Mak CM (2012). BAG3-related myofibrillar myopathy in a Chinese family. Clin Genet.

[CR31] Li H, Durbin R (2009). Fast and accurate short read alignment with Burrows-Wheeler transform. Bioinformatics.

[CR32] Lu S, Horowits R (2008). Role of nonmuscle myosin IIB and N-RAP in cell spreading and myofibril assembly in primary mouse cardiomyocytes. Cell Motil Cytoskelet.

[CR33] Lu S, Carroll SL, Herrera AH, Ozanne B, Horowits R (2003). New N-RAP-binding partners alpha-actinin, filamin and Krp1 detected by yeast two-hybrid screening: implications for myofibril assembly. J Cell Sci.

[CR34] Luo G, Leroy E, Kozak CA, Polymeropoulos MH, Horowits R (1997). Mapping of the gene (NRAP) encoding N-RAP in the mouse and human genomes. Genomics.

[CR35] Luo G, Zhang JQ, Nguyen TP, Herrera AH, Paterson B, Horowits R (1997). Complete cDNA sequence and tissue localization of N-RAP, a novel nebulin-related protein of striated muscle. Cell Motil Cytoskelet.

[CR36] Luo G, Herrera AH, Horowits R (1999). Molecular interactions of N-RAP, a nebulin-related protein of striated muscle myotendon junctions and intercalated disks. Biochemistry.

[CR37] Manisastry SM, Zaal KJ, Horowits R (2008). Myofibril assembly visualized by imaging N-RAP, alpha-actinin, and actin in living cardiomyocytes. Exp Cell Res.

[CR38] McGrath MJ, Cottle DL, Nguyen MA, Dyson JM, Coghill ID, Robinson PA, Holdsworth M, Cowling BS, Hardeman EC, Mitchell CA, Brown S (2006). Four and a half LIM protein 1 binds myosin-binding protein C and regulates myosin filament formation and sarcomere assembly. J Biol Chem.

[CR39] Mohiddin SA, Lu S, Cardoso JP, Carroll S, Jha S, Horowits R, Fananapazir L (2003). Genomic organization, alternative splicing, and expression of human and mouse N-RAP, a nebulin-related lim protein of striated muscle. Cell Motil Cytoskelet.

[CR40] Ng EK, Lee SM, Li HY, Ngai SM, Tsui SK, Waye MM, Lee CY, Fung KP (2001). Characterization of tissue-specific LIM domain protein (FHL1C) which is an alternatively spliced isoform of a human LIM-only protein (FHL1). J Cell Biochem.

[CR41] Odgerel Z, Sarkozy A, Lee HS, McKenna C, Rankin J, Straub V, Lochmüller H, Paola F, D’Amico A, Bertini E, Bushby K, Goldfarb LG (2010). Inheritance patterns and phenotypic features of myofibrillar myopathy associated with a BAG3 mutation. Neuromuscul Disord.

[CR42] Pfeffer G, Barresi R, Wilson IJ, Hardy SA, Griffin H, Hudson J, Elliott HR, Ramesh AV, Radunovic A, Winer JB, Vaidya S, Raman A, Busby M, Farrugia ME, Ming A, Everett C, Emsley HC, Horvath R, Straub V, Bushby K, Lochmüller H, Chinnery PF, Sarkozy A (2014). Titin founder mutation is a common cause of myofibrillar myopathy with early respiratory failure. J Neurol Neurosurg Psychiatry.

[CR43] Quinzii CM, Vu TH, Min KC, Tanji K, Barral S, Grewal RP, Kattah A, Camaño P, Otaegui D, Kunimatsu T, Blake DM, Wilhelmsen KC, Rowland LP, Hays AP, Bonilla E, Hirano M (2008). X-Linked dominant scapuloperoneal myopathy is due to a mutation in the gene encoding four-and-a-half-LIM protein 1. Am J Hum Genet.

[CR44] Rosati A, Graziano V, De Laurenzi V, Pascale M, Turco MC (2011). BAG3: a multifaceted protein that regulates major cell pathways. Cell Death Dis.

[CR45] Ruparelia AA, Oorschot V, Vaz R, Ramm G, Bryson-Richardson RJ (2014). Zebrafish models of BAG3 myofibrillar myopathy suggest a toxic gain of function leading to BAG3 insufficiency. Acta Neuropathol.

[CR46] Schessl J, Zou Y, McGrath MJ, Cowling BS, Maiti B, Chin SS, Sewry C, Battini R, Hu Y, Cottle DL, Rosenblatt M, Spruce L, Ganguly A, Kirschner J, Judkins AR, Golden JA, Goebel HH, Muntoni F, Flanigan KM, Mitchell CA, Bönnemann CG (2008). Proteomic identification of FHL1 as the protein mutated in human reducing body myopathy. J Clin Investig.

[CR47] Schessl J, Taratuto AL, Sewry C, Battini R, Chin SS, Maiti B, Dubrovsky AL, Erro MG, Espada G, Robertella M, Saccoliti M, Olmos P, Bridges LR, Standring P, Hu Y, Zou Y, Swoboda KJ, Scavina M, Goebel HH, Mitchell CA, Flanigan KM, Muntoni F, Bönnemann CG (2009). Clinical, histological and genetic characterization of reducing body myopathy caused by mutations in FHL1. Brain.

[CR48] Schessl J, Columbus A, Hu Y, Zou Y, Voit T, Goebel HH, Bönnemann CG (2010). Familial reducing body myopathy with cytoplasmic bodies and rigid spine revisited: identification of a second LIM domain mutation in FHL1. Neuropediatrics.

[CR49] Schmieder R, Edwards R (2011). Quality control and preprocessing of metagenomic datasets. Bioinformatics.

[CR50] Schoser B, Goebel HH, Janisch I, Quasthoff S, Rother J, Bergmann M, Müller-Felber W, Windpassinger C (2009). Consequences of mutations within the C terminus of the FHL1 gene. Neurology.

[CR51] Selcen D, Engel AG (2004). Mutations in myotilin cause myofibrillar myopathy. Neurology.

[CR52] Selcen D, Engel AG (2005). Mutations in ZASP define a novel form of muscular dystrophy in humans. Ann Neurol.

[CR53] Selcen D, Muntoni F, Burton BK, Pegoraro E, Sewry C, Bite AV, Engel AG (2009). Mutation in BAG3 causes severe dominant childhood muscular dystrophy. Ann Neurol.

[CR54] Shalaby S, Hayashi YK, Goto K, Ogawa M, Nonaka I, Noguchi S, Nishino I (2008). Rigid spine syndrome caused by a novel mutation in four-and-a-half LIM domain 1 gene (FHL1). Neuromuscul Dis.

[CR55] Sheikh F, Bang ML, Lange S, Chen J (2007). ‘Z’eroing in on the role of cypher in striated muscle function, signaling, and human disease. Trends Cardiovasc Med.

[CR56] Sheikh F, Raskin A, Chu PH, Lange S, Domenighetti AA, Zheng M, Liang X, Zhang T, Yajima T, Gu Y, Dalton ND, Mahata SK, Dorn GW, Brown JH, Peterson KL, Omens JH, McCulloch AD, Chen J (2008). An FHL1-containing complex within the cardiomyocyte sarcomere mediates hypertrophic biomechanical stress responses in mice. J Clin Invest.

[CR57] Tullio AN, Accili D, Ferrans VJ, Yu ZX, Takeda K, Grinberg A, Westphal H, Preston YA, Adelstein RS (1997). Nonmuscle myosin II-B Is required for normal development of the mouse heart. Proc Natl Acad Sci USA.

[CR58] van Blitterswijk M, van Es MA, Hennekam EA, Dooijes D, van Rheenen W, Medic J, Bourque PR, Schelhaas HJ, van der Kooi AJ, de Visser M, de Bakker PI, Veldink JH, van den Berg LH (2012). Evidence for an oligogenic basis of amyotrophic lateral sclerosis. Hum Mol Genet.

[CR59] Vasilopoulos Y, Cork MJ, Teare D, Marinou I, Ward SJ, Duff GW, Tazi-Ahnini R (2007). A nonsynonymous substitution of cystatin A, a cysteine protease inhibitor of house dust mite protease, leads to decreased mRNA stability and shows a significant association with atopic dermatitis. Allergy.

[CR60] Vicart P, Caron A, Guicheney P, Li Z, Prévost MC, Faure A, Chateau D, Chapon F, Tomé F, Dupret JM, Paulin D, Fardeau M (1998). A missense mutation in the alphaB-crystallin chaperone gene causes a desmin-related myopathy. Nat Genet.

[CR61] Vorgerd M, van der Ven PF, Bruchertseifer V, Löwe T, Kley RA, Schröder R, Lochmüller H, Himmel M, Koehler K, Fürst DO, Huebner A (2005). A mutation in the dimerization domain of filamin c causes a novel type of autosomal dominant myofibrillar myopathy. Am J Hum Genet.

[CR62] Wang K, Li M, Hakonarson H (2010). ANNOVAR: functional annotation of genetic variants from high-throughput sequencing data. Nucleic Acids Res.

[CR63] Wang L, Miao J, Li L, Wu D, Zhang Y, Peng Z, Zhang L, Yuan Z, Sun K (2013). Identification of an FHL1 protein complex containing gamma-actin and non-muscle myosin IIB by analysis of protein-protein interactions. PLoS One.

[CR64] Windpassinger C, Schoser B, Straub V, Hochmeister S, Noor A, Lohberger B, Farra N, Petek E, Schwarzbraun T, Ofner L, Löscher WN, Wagner K, Lochmüller H, Vincent JB, Quasthoff S (2008). An X-linked myopathy with postural muscle atrophy and generalized hypertrophy, termed XMPMA, is caused by mutations in FHL1. Am J Hum Genet.

[CR65] Zarraga IG, Zhang L, Stump MR, Gong Q, Vincent GM, Zhou Z (2011). Nonsense-mediated mRNA decay caused by a frameshift mutation in a Large kindred of type 2 long QT syndrome. Heart Rhythm.

